# Current Disease Threats for Cultivated Crab *Eriocheir sinensis* in China

**DOI:** 10.1155/2023/3305963

**Published:** 2023-04-03

**Authors:** Zhengfeng Ding

**Affiliations:** Institute of Aquatic Biology and Jiangsu Key Laboratory for Biofunctional Molecules, College of Life Sciences and Chemistry, Jiangsu Second Normal University, 77 West Beijing Road, Nanjing 210013, China

## Abstract

The Chinese mitten crab, *Eriocheir sinensis*, is a commercially important crustacean in China due to its great commercial value and compatibility in a variety of aquaculture systems. However, increases in its production have been accompanied by the emergence of various diseases affecting yield, profit, and trading potential. In this study, we review the pathogenic agents associated with *E. sinensis* since the start of its commercial culture. The history of crab cultivation implies that increased pathogen transfer can occur as *E. sinensis* aquaculture grows because polyculture of *E. sinensis* with other aquaculture species is a prevalent practice. With this in mind, a special focus of this review is placed on pathogens that were initially discovered in other crustacean species but have since been demonstrated to infect and cause disease in *E. sinensis*. We expect that this review will not only offer recommendations for disease management in the *E. sinensis* aquaculture sector but will also advance other crustacean cultivation.

## 1. Introduction

Aquaculture is the fastest growing agricultural sector in the world, and more than 87 million tons of fish, molluscs, and crustaceans were produced worldwide through aquaculture in 2020, making up just under half of the total production [1]. Therein, crustacean aquaculture constitutes a significant portion of the current expansion and is anticipated to contribute a greater total in the future. In addition to being a significant supply of aquatic dietary protein, crustaceans are also a significant source of income for those involved in the value chain. Their trade and aquaculture are crucial for developing countries, such as crab in China in particular, which is now the most commercially traded fishery in terms of value [2].

The Chinese mitten crab, *Eriocheir sinensis* (H. Milne Edwards), is an economically significant species of crustacean in China. However, it has gained notoriety as an invasive species in Europe and North America after probably migrating there via ship ballast water over the past century [3]. The culture of crab dates to the 1960s, when changes in hatchery methods were first developed in response to reductions in wild crab catches in China as a result of overfishing and the engineering of impassable waterways. Large-scale production has become feasible due to improved hatchery rearing techniques, and the annual return of *E. sinensis* in China increased from 17,500 t in 1993 to 796,535 t in 2014 [4]. By 2018, 8% of global crustacean aquaculture production was made up of *E. sinensis* behind white-legged shrimp, *Penaeus vannamei* and red swamp crayfish, *Procambarus clarkii* at 53% and 18%, respectively [5].

However, there have recently been a number of concerns regarding the sustainability of *E. sinensis* aquaculture. Similar to shrimp or prawn farm operations, when new aquaculture techniques appear at the same time as a concentrated push to boost crab farming production, the danger of crab disease also increases [6]. Numerous diseases, induced by bacterial, viral, fungal, and other eukaryotic pathogens, have been found in cultivated *E. sinensis*. These pathogens can cause mortality in crabs or result in the production with a low market value. In this review, we explain the disease-causing pathogens that have affected *E. sinensis* since this species was first commercially cultivated. We also summarize the lessons acquired from prior disease outbreaks and discuss how culture methods can reduce disease transmission in *E. sinensis* and other commercially valuable crustaceans.

## 2. Bacteria

### 2.1. Spiroplasma

A review of the related literature reveals that *Spiroplasma* sp., the first spiroplasma to be isolated from the crab *E. sinensis*, is the subject of most studies. An overview of the spiroplasma disease is provided in Table 1.

*Spiroplasma* has historically mainly infected plants, insects, and ticks [7]. However, a spiroplasma associated with highly pathognomonic symptoms of tremor disease (TD) was isolated from the crab *E. sinensis* [8]. Uncontrollable shaking of the pereiopods was the most obvious symptom of sick crabs, and this led farmers to refer to it as “tremor disease” [9]. Further investigation revealed that the pathogen's presence in the thoracic ganglion and myoneural junctions likely affects nerve transmission, resulting in the pereiopods' distinctive paroxysmal tremors. The agent was exceptional in that it had a helical morphology, rotating motility, passed through membrane filters as small as 0.22 *μ*m, and could be grown in liquid media *in vitro*. It could cause systemic inflammatory reactions, resulting in weakness and rapid death while being removed from traps [9].

In addition to the 16S rRNA gene sequence analysis, characteristics such as the absence of a cell wall, helical morphology, motility, and in vitro culture have been confirmed, and the pathogen was officially named *Spiroplasma eriocheiris* sp. nov [10].

Since this initial discovery, the spiroplasma pathogen has also been found in other cultivated crustaceans, including the crayfish *Procambarus clarkii* [11] and shrimp *Macrobrachium nipponense* [12] that typically inhabited the same pond as *E. sinensis*. Furthermore, it has been observed in a marine or brackish shrimp *Penaeus vannamei* [13] and in sediments around susceptible hosts [14]. The clinical signs of the *P. vannamei* included expanded chromatophores and the farmers referred to as “standing shrimp syndrome,” in which floating dead shrimp appeared to be balancing on their tails, looking at the sky [13]. It could be very pathogenic to crayfish *P. clarkii*, with the result of an uncontrollable shaking of the pereiopods as well [11]. However, in shrimp *M. nipponense*, tremors of the pereiopod was not observed, but quick death was often observed while being removed from the traps [12]. The studies published above indicate that the crab and other crustacean aquaculture sectors could be severely threatened by the widespread occurrence of spiroplasma in the aquatic environment.

Epidemiological studies and diagnosis are important in the fight against disease. A variety of techniques have been developed to successfully detect spiroplasma in the crab *E. sinensis*, including clinical signs, light microscopy, electron microscopic histopathology [10], *in situ* hybridization [15], PCR-based molecular diagnostics, SYBR Green *q*PCR [16], enzyme-linked immunosorbent assays [17], and an immunochromatographic strip test using gold nanoparticles [18].

According to the assumption, *S. eriocheiris* infected crabs had reduced resistance due to compromised immunity, resulting in death. Hence, knowing the defense mechanism of *E. sinensis* and their immune responses to *S. eriocheiris* has become a priority [17, 18]. Numerous studies on the immune reactions of *E. sinensis* to spiroplasma infection have been published. This field has advanced significantly in recent years, especially with the current development of high-throughput methodologies for global data acquisition, computational tools for data analysis, data storage, and mathematical algorithms. Technologies known as “omics” have recently surfaced to offer a comprehensive view of all cellular components and their interactions. The majority of the research was performed on hemocytes because they were the first cells that spiroplasma targeted in *E. sinensis* [10]. Using a deep sequencing method, Ou et al. provided the miRNAs in *E. sinensis* and their altered expression in response to spiroplasma infection. Numerous miRNAs showed significant differences in their levels between healthy and infected hemocytes, supporting the idea that some miRNAs may be crucial for spiroplasma-crab interactions [19]. Meng et al. performed a proteomic analysis of the effects of spiroplasma on hemocytes of *E. sinensis* and quantified 76 differentially expressed proteins using the isobaric tag for relative and absolute quantization (iTRAQ) [20]. Furthermore, the interaction between spiroplasma and crab hemocytes was suggested to involve numerous biological processes and pathways by bioinformatic analysis of the ubiquitination of differently expressed proteins, including the ubiquitin system, endocytosis, prophenoloxidase system (proPO system), cell apoptosis, and glycolysis [21]. Additional findings supported that crab ability to combat spiroplasma in the thoracic ganglia involved the proPO system, Wnt signaling pathway, and processes related to Ca^2+^ regulation [22]. Specific immune-related genes of *E. sinensis*, including extracellular copper/zinc superoxide dismutase (ecCuZnSOD) [23], cathepsin D [24], calcium/calmodulin-dependent protein kinase II [25], ShK-domain serine protease [26], and adhesin-like protein ALP41 [27], were also identified and confirmed to play important roles upon spiroplasma infection.

The aforementioned discoveries have undoubtedly improved our understanding of the crab's immune response to spiroplasma and may make it easier to create new preventive measures to guard against bacterial infection in crustaceans.

### 2.2. Vibrios

Vibrios such as *Vibrio parahaemolyticus*, *Vibrio anguillarum*, and *Vibrio alginolyticus* are frequently found in hatcheries and farms of *E. sinensis* [28, 29]. The spine damage and the poor appetite that crab larvae typically experience lead to high mortality rates when they are infected with vibrios. Additionally, between 2003 and 2006, water samples and crabs from the River Thames in the United Kingdom were tested for *V. parahaemolyticus*. During this testing period, *V. parahaemolyticus* was positively detected in all samples. Compared to winter, *V. parahaemolyticus* levels were higher in summer. The authors proposed that *V. parahaemolyticus* might selectively colonize the hepatopancreas of *E. sinensis* by preferential adhesion in the gut of the crab [30]. Similarly, *V. parahaemolyticus* was detected in three crabs purchased from a variety of markets in China and six crabs were exported to Japan. These crabs were cultivated close to Yangcheng Lake in Suzhou city of China, a large freshwater lake that is well known for being the location of crab production [31]. We should be aware of this widely spread bacterium, which can cause or produce bacterial diseases in freshwater and marine environments, although there have only been a few recent reports of vibrio-infection in naturally cultured *E. sinensis*.

## 3. Virus

Crab viruses are the first crustacean viruses to be discovered in decapods [32]. However, only a few have been reported from freshwater crabs, while the majority was connected to marine crabs. Under cultivation, viral pathogens appear to have the most significant effect on the growth and survival of crabs, including *E. sinensis*.

### 3.1. White Spot Syndrome Virus (WSSV)

#### 3.1.1. Natural Infection of WSSV with *E. sinensis*

**Figure 1:**
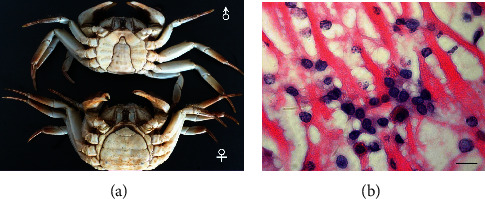
Natural infection of WSSV in the cultivated crab *Eriocheir sinensis* in China, and the infected crabs exhibited signs of weakness and a weak response to stimulation. White spots in the cuticle and reddish body color were not observed [35]. (a) Naturalinfection of WSSV in the cultivated crab *Eriocheir sinensis* in China. (b) Evident signs of WSSV infection showing widespread hypertrophied nuclei of infected cells and scale bar = 50 *μ*m (H&E staining).

The white spot disease (WSS), which was brought on by the white spot syndrome virus (WSSV), has emerged as the biggest threat to the world's aquaculture of crustaceans [33]. The International Committee on Virus Taxonomy placed the double-stranded DNA virus in its own brand-new genus, *Whispovirus*, within the *Nimaviridae* family [34]. Ding et al. first observed a WSSV epidemic in crab *E. sinensis* with a high mortality rate in China. The target tissues, including the gills, cardiac muscle, intestinal tract, and testes, were severely infected with WSSV in all moribund crabs. The crabs did not exhibit the obvious gross symptoms of WSSV infection that were present in penaeid shrimp (white spots in the cuticle and reddish body color) (Figures 1(a) and 1(b)). This may be due to the thickness of the cuticle and its dark color, which is caused by the melanin pigment [35].

Additionally, the presence of WSSV in the freshwater crayfish *Procambarus clarkii*, which has been shown to be particularly supportive of WSSV replication, was also confirmed by additional samples taken from the same ponds as *E. sinensis* [35]. *P. clarkii* may be acting as a virus carrier, helping to maintain WSSV in the environment. Given that 98 species of crustaceans have been identified as hosts or carriers of WSSV [36], it is important to pay close attention to any potential horizontal transmission of the virus between *E. sinensis* and other species. Farmers should be aware of the ever-expanding list of potential WSSV carriers and take precautions to keep their crabs out of contact with the carriers and away from the same waterways throughout the entire cultivation process, especially in regions where they are grown close together.

#### 3.1.2. Interaction between WSSV and *E. sinensis*

An emerging research focus is the molecular mechanism underlying the interaction between *E. sinensis* and WSSV. Ding et al. provided information on the temporal and spatial dynamics of WSSV in the crab. The stomach was the most popular tissue for WSSV replication in the early stages of infection, and the severity of infection throughout the process was highest in the gills, pleopods, and stomach [37]. Similarly, the tissue tropism analysis of both experimentally infected and wild-caught crabs reveals that WSSV primarily targets tissues derived from the ectoderm and mesoderm, particularly the cuticular epithelium and subcuticular connective tissues [38]. Therefore, the tissues of gills, stomach, hemolymph, pleopods, or abdominal muscle are advised in combination for WSSV diagnosis in *E. sinensis*.

It was discovered that WSSV increased virus replication during infection by utilizing crab microRNA-7. MicroRNA-7 has the crab myeloid differentiation factor 88 (Myd88) as its target gene [39]. In addition, following the discovery of three novel Spätzle genes in *E. sinensis*, further investigation established their role in crab innate immune defense against WSSV by activating the expression of antimicrobial peptides [40]. These investigations added to our understanding of the crab antiviral immune defense.

The impact of the gut microbiome on the host-pathogen interaction was also considered essential for disease prevention. On the MiSeq Illumina sequencing platform, a 16S rRNA approach was used to investigate the dynamics of gut bacterial communities in the crab *E. sinensis* that was challenged with WSSV. Four phyla (*Firmicutes*, *Proteobacteria*, *Tenericutes*, and *Bacteroidetes*) predominate. At various stages of WSSV infection, there were significant differences in the abundance of over 12 bacterial phyla, 44 orders, and 68 families [41]. The study found that changes in the intestinal microbiome were closely associated with the severity of WSSV infection and that these indicative taxa could be used to assess crab health status.

#### 3.1.3. WSSV Control

The description here was added because the WSSV control practice offers the single most striking example of disease control in *E. sinensis* cultivation. In the same way that penaeid shrimp have been shown to be more susceptible to WSSV after molting and that wounding is a major contributing factor that facilitates WSSV infection [42], it is possible that the crab *E. sinensis* may be susceptible to WSSV when molting, when weak, or by some other kind of stress. In environments where fighting or cannibalism is common, especially in conditions of higher density rearing, wounding may increase transmission. Therefore, it may be advised to stock fewer crabs in larger ponds.

Prevention is widely accepted as the best way to prevent outbreaks of WSSV. Several studies have examined how disinfectants affect WSSV [43]. A vaccine approach to the management of WSSV has also been investigated [44]. Moreover, there have been suggested biosecurity measures to keep the pathogen out or lessen its risk [45]. The crab industry might also benefit from measures such as the stocking of larvae that have been PCR-verified to be WSSV-negative, the use of disinfectants, closed culture systems with reduce water exchange, bird scares, crab fences, foot/tire baths, and restricted access to the farm.

Recent reports of several medicinal plants or active substances derived from plants that have anti-WSSV activity highlight the potential of herbal remedies to be effective treatments for WSSV. Based on a viral infection model in *E. sinensis*, Chen et al. chose 30 herbal remedies and selected *Ophiopogon japonicus* (also known as Mai Dong in Chinese, distributed primarily in the southwest areas of China) as having the strongest inhibitory activity against WSSV. Treatment with *O. japonicus* can prevent WSSV replication in early stages and increase autophagy and antioxidative activity in *E. sinensis* [46]. Moreover, several studies raised the possibility of using Poaceae plant species, *Cynodon dactylon,* to combat WSSV infection. HPLC-DAD was used to confirm the presence of some antiviral compounds in *C. dactylon*. The *in vivo* challenge test revealed that ethanolic extracts of *C. dactylon*, at concentrations of 100 to 150 mg/kg, can shield crustaceans from WSSV infection [47, 48].

### 3.2. Reovirus

A new reovirus (EsRV905) that was 55 nm in diameter, icosahedral, nonenveloped, and had a mean buoyant density of 1.39 g/cm^3^ in a CsCl gradient was isolated from *E. sinensis* in Wuhan, China. Twelve dsRNA fragments make up the genome, and their electrophoretic patterns are 3/4/2/3. The gills, digestive system, and hepatopancreas were among the tissues targeted. The virus may differ from other crab reoviruses and represent a new genus of Reoviridae. Only 30% of experimentally infected *E. sinensis* died from the prepurified reovirus, but the virus can still be detected in surviving crabs [49].

### 3.3. Ronivirus

The crab *E. sinensis* was found to have a disease known as “sigh disease” due to apparent respiratory issues. A roni-like virus with the typical traits of Roniviridae was found in a number of tissues, including the intestine, cardiac muscle, hepatopancreas, and gills. The infection was characterized by pyknosis, karyorrhexis, and the formation of inclusion-like structures, which corresponded to multifocal to generalized necrosis. Surface spikes covered the virions, but nucleocapsids were not readily visible. The mortality could reach 100% under test conditions [50].

## 4. Parasites

### 4.1. Microsporidia

**Figure 2:**
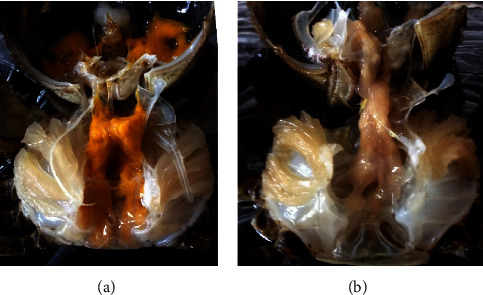
Gross signs of the *Hepatospora eriocheir* infected the crab *Eriocheir sinensis*. (a) Healthy crabs with the hepatopancreas that are golden-yellow; (b) infected crabs that transform the color of the hepatopancreas into virtually white (from Ding et al., journal of fish diseases, 40 (7): 919–927) [52].

Microsporidia are tiny obligatory intracellular parasites initially regarded as primitive eukaryotic protozoa but are recently reclassified as fungi. They are known to infect aquatic hosts in nearly half of their genera but are likely widespread and significantly underreported in aquatic environments [51]. Recent severe microsporidian outbreaks in Jiangsu Province, China, resulted in catastrophic loss of the crab *E. sinensis*. A notable sign of tissue-specific infection was that the color of the hepatopancreas changed from golden yellow to nearly white (Figures 2(a) and 2(b)). There is evidence that the disease is closely related to the microsporidia *Hepatospora eriocheir* [53]. *Hepatospora* spp. has been isolated from different crustacean hosts, inhabiting different habitats and niches: the Chinese mitten crab (*E. sinensis*), the pea crab (*Pinnotheres pisum*), and the marine edible crab (*Cancer pagurus*) [54]. However, to our knowledge, *H. eriocheir* was the only microsporidia parasite found in *E. sinensis*.

According to histological findings, the mitochondria of the hepatopancreas cells were located close to the plasmalemma of *H. eriocheir* meronts, likely to facilitate ATP uptake directly from the crab host. Transcriptome data also suggested that the resulting hepatopancreas damage and subsequent siphoning of crab energy resources may have a significantly negative impact on the metabolic and nutritional pathways of hepatopancreas cells. Additionally, infection with *H. eriocheir* significantly changed the expression of genes involved in “starch and sucrose metabolism,” “glycan degradation,” “fatty acid biosynthesis,” and “amino acid metabolism” [55]. These results demonstrated the high energy cost of microsporidian infection in *E. sinensis*.

Given that the hepatopancreas of *E. sinensis* served as a hub for lipid metabolism and energy production, the comparative lipid metabolism profiles were further examined using a lipidomics approach based on liquid chromatography mass spectrometry (LC-MS) [56]. According to the results, microsporidia *H. eriocheir* caused apparent changes in the lipid phenotype of the hepatopancreas. The significant alteration of 67 lipids allowed the identification of these molecules as useful biomarkers. The majority was made up of triglycerides (TG) and diglyceride (DG). In particular, more than 94% of the distinguished lipids showed a similar modified trend with significantly lower contents, implying blatant energy exploitation of the parasite. These findings suggest that *H. eriocheir* can “starve” the crab by destroying hepatopancreas tissue together with the necessary host energy sources, causing the corresponding alterations in lipid metabolism and a loss of hepatopancreatic color (Figures 2(a) and 2(b)) [56].

### 4.2. Yeast

The “milky disease” of *E. sinensis* was recently linked to the yeast pathogen *Metschnikowia bicuspidata* in northern China. Weakness, opaque or whitish muscles, and milky hemolymph are the clinical symptoms. All major tissues, including the hemolymph, hepatopancreas, muscle, heart, and gills, contained large numbers of *M. bicuspidata*, indicating that yeast infection probably spreads through the circulatory system [57]. According to proteomics analyses, downregulated proteins showed that the yeast inhibited crab hemocyte regeneration and hemolymph agglutination, while up-regulated proteins were involved in the phenoloxidase, phagocytosis, and ROS systems [58].

### 4.3. Polyascus gregaria

*Polyascus gregaria*, a parasite of the crab *E. sinensis*, has recently been observed in several Chinese provinces in the lower Yangtze River, including Jiangsu, Zhejiang, and Shanghai. This species of parasitic barnacle exhibits highly specialized and wildly divergent forms that almost entirely lack the typical characteristics of crustaceans during adult life stages. The parasite has internal and external parts that are used for nutrition and reproduction, respectively. In order to absorb the nutrition, the internae form rootlets and spread like a tree through a variety of crab tissues, including the hepatopancreas, gills, and muscles. The abdomen is where the externae, which have reproductive properties, are attached. Although externae erupt in freshwater during parasitism, reproduction occurs only when the host returns to the estuary, where the larvae are then born and released. In both males and females, the maximum prevalence could rise above 50% in June and gradually declined from July to October [59].

Numerous studies have examined the immunological mechanism of *E. sinensis* following *P. gregaria* infection. Through the TMT analysis, Yang et al. investigated the male crab's innate immune response to *P. gregaria* infection. The selection of differentially expressed proteins from hepatopancreas tissues revealed 352 up-regulated proteins and 246 downregulated proteins, including ATG, serpin, iron-related protein, integrin, cathepsin, a member of the Rab family, and lectin [59]. Additionally, while the phenoloxidase activity was significantly reduced in infected *E. sinensis*, it was significantly increased in superoxide dismutase, acid phosphatase, and alkaline phosphatase activities. The metabolomic profiling revealed that the differential metabolites were mostly enriched in the glycerophospholipid pathway, suggesting significant structural and functional abnormalities in the membranes of the hepatopancreas. Long-term *P. gregaria* infection may silence the innate immune system, disturb intracellular homeostasis, and lead to immune-related dysfunction of the cell membrane in the crab [60].

To control parasitic infections, new techniques should be developed that target the vulnerable life stages of each individual parasitic species. Because they have such a high capacity for environmental adaptation, it is also important to understand that a single strategy is rarely sufficient. Integrated strategies to combat parasitic diseases should be promoted critically.

## 5. Discussion

Although the aquaculture industry of *E. sinensis* has grown significantly in recent decades, infectious diseases are now a barrier to further intensification. Diseases are spread in a variety of unpredictable ways but are primarily spread through the careless or illegal transboundary movement of diseased crab stocks from one region to another or by uninfected exotic stocks moving to a new region where they become infected with local pathogens, which may have gone unnoticed previously because they did not affect local species. Exclusion from the aquaculture system is the current primary control strategy for the majority diseases [6]. This necessitates the strictest doable biosecurity measures being put in place, along with ongoing vigilance for both new and existing pathogens. Practical, affordable, and approved therapeutic approaches are desperately needed, but they are still lacking for *E. sinensis*, particularly in grow-out ponds.

Monitoring of crab stock development, fry production, and juvenile grow-out requires quick, sensitive, and targeted pathogen detection methods. The best approaches must be point-of-care (POC) approaches (methods that are simple enough for farmers themselves to apply and interpret in actual production facilities such as hatcheries and farms) [61]. They allow management decisions to be made in the present without having to expect a delayed test result from a distant laboratory. Next-generation sequencing techniques (such as metagenomics) allow profiling of microbial communities in crabs, in addition to screening for known and unidentified pathogens. Understanding the cooperative and competitive interactions that may exist between pathogens and resident microbes may benefit from this information. The data collected could be used to create more affordable disease control strategies.

New and potential areas of research for improving crab disease control and stabilization include the use of “vaccination” with pathogen- and host-derived molecules, the selective activation of variable host antimicrobial responses, the use of host-derived antimicrobial peptides, the use of general immunostimulants as feed additives, the addition of double-stranded RNA in the feed to induce the host-RNA interference mechanism against viruses, and the selection or induction of endogenous viral elements (EVE) to provide the crab with heritable tolerance to viral infections [61]. As potential alternatives to the use of antibiotics in aquaculture, probiotics, prebiotics, their combinations (synbiotics), nonviable bacterial or metabolic byproducts derived from probiotic bacteria, plant-derived natural compounds, bacteriophages, and interference of quorum sensing can also be considered. It is particularly important that an innovative and leading-edge tool, nanotechnology, has a broad range of applications and potential for controlling disease in *E. sinensis* cultivation [62]. Improvements in engineering and biotechnology will be necessary, and many of the cutting-edge disease prevention and treatment strategies that are currently being researched will become commonplace. In addition, the continuous support of research by pertinent experts working in multidisciplinary teams, as well as the education of college students, aquaculture practitioners, and consumers, will be essential to achieve these goals.

## Figures and Tables

**Table 1:** An overview of the spiroplasma disease in cultivated crustaceans.

Hosts	Global locations	Clinical signs of infection	Histopathological signs of infection	References
Freshwater crab, *Eriocheir sinensis*	Anhui and Jiangsu provinces, China	Weakness, anorexia, intense paroxysmal tremors, and death	Transverse binary division, formation of inclusion bodies or appearance as single cells in the cytoplasm, and possible inhibition of phagosome-lysosome fusion	[9, 10]
Freshwater crayfish, *Procambarus clarkii*	Jiangsu province of China	Weakness, appendage tremor, and death	Hemocytic congestion, hemocytic nodule formation, phagocytosis (sometimes accompanied by melanization), nucleus enlargement, and epithelial cell shedding	[11, 15]
Freshwater prawns, *Macrobrachium rosenbergii*	Gaoyou county, Jiangsu provinces of China	Aggregation at the side of ponds and rapid death when seining	Spiroplasmas reproduced within the inclusion in hemocytes, cardiac muscle, and connective tissues	[10]
River prawn, *Macrobrachium nipponense*	Baoying, Jiangsu province, China	Weakness, anorexia, and death; cultured in the same pond with *E. sinensis* exhibiting tremor symptom	Numerous spiroplasmas were observed within the inclusion bodies of hemocytes	[12]
Marine shrimp, *Penaeus vannamei*	A Colombian shrimp farm located on the Caribbean coast	Referred as “standing shrimp syndrome:” the floating, dead shrimp appeared to be balancing on their tails, looking at the sky	Multifocal, moderate to severe, systemic inflammatory reactions in the form of hemocytic congestion, hemocyte coagulation into loose clots, hemocytic nodule formation, phagocytosis, and fibrosis	[13]

## Data Availability

Data sharing is not applicable to this article as no datasets were generated or analyzed during the current study.
